# How the clinical dosage of bone cement biomechanically affects adjacent vertebrae

**DOI:** 10.1186/s13018-020-01906-0

**Published:** 2020-08-31

**Authors:** Xu-shi Chen, Jian-ming Jiang, Pei-dong Sun, Zhao-fei Zhang, Hai-long Ren

**Affiliations:** 1 Department of Spinal Surgery, Huizhou Municipal Central Hospital, Huizhou, Guangdong China; 2 Department of Spine Surgery, Nanfang Hospital, Southern Medical University, Guangzhou, Guangdong China; 3 Guangdong Provincial Key Laboratory of Medical Biomechanics, School of Basic Medical Sciences, Southern Medical University, Guangzhou, Guangdong China; 4 Department of Orthopedic Surgery, Guangzhou Hospital of Integrated Traditional and Western Medicine, 87 Yingbin Road, Huadu District, Guangzhou, Guangdong China

**Keywords:** Osteoporotic vertebral compression fracture, Percutaneous vertebroplasty, Stiffness, Strain

## Abstract

**Objective:**

This study evaluated the biomechanical changes in the adjacent vertebrae under a physiological load (500 N) when the clinically relevant amount of bone cement was injected into fractured cadaver vertebral bodies.

**Methods:**

The embalmed cadaver thoracolumbar specimens in which each vertebral body (T12–L2) had a BMD of < 0.75 g/cm^2^ were used for the experiment. For establishing a fracture model, the upper one third of the L1 vertebra was performed wedge osteotomy and the superior endplate was kept complete. Stiffness of specimens was measured in different states. Strain of the adjacent vertebral body and intervertebral disc were measured in pre-fracture, post-fracture, and after augmentation by non-contact optical strain measurement system.

**Results:**

The average amount of bone cement was 4.4 ml (3.8–5.0 ml). The stiffness of after augmentation was significantly higher than the stiffness of post-fracture (*p* < 0.05), but still lower than pre-fracture stiffness (*p* < 0.05). After augmentation, the adjacent upper vertebral strain showed no significant difference (*p* > 0.05) with pre-fracture, while the strain of adjacent lower vertebral body was significantly higher than that before fracture (*p* < 0.05). In flexion, T12/L1 intervertebral disc strain was significantly greater after augmentation than after the fracture (*p* < 0.05), but there was no significant difference from that before the fracture (*p* > 0.05); L1/2 vertebral strain after augmentation was significantly less than that after the fracture (*p* < 0.05), but there was no significant difference from that before the fracture (*p* > 0.05).

**Conclusions:**

PVP may therefore have partially reversed the abnormal strain state of adjacent vertebral bodies which was caused by fracture.

## Introduction

Percutaneous vertebroplasty (PVP) was an effective treatment for patients with osteoporotic vertebral compression fractures when conservative treatment is ineffective. Clinical studies have shown, however, that new vertebral compression fractures occur in some patients who have undergone PVP. The reported incidence of these new vertebral compression fractures (including both adjacent and non-adjacent segments) after PVP has ranged widely (5.5–33.5%), with compression fractures of adjacent segments becoming more common [1–3]. Most authors attributed the new fractures to the changes in spinal biomechanical properties after PVP [4, 5], although some clinical studies found that patients undergoing conservative treatment of an adjacent vertebral fracture were more likely to experience new fractures than those who had undergone PVP [6].

Biomechanical experimental results confirmed that a vertebral body reinforced by bone cement could cause local biomechanical changes, and some clinicians have begun to carry out prophylactic adjacent-segment vertebroplasty [7–9]. The conclusions, however, were not consistent among the various biomechanical studies because of differences in the fracture model, amount and type of bone cement, and other factors. We found that the amount of bone cement in many of the earlier biomechanical experiments was far greater than the clinically relevant amount. We also observed clinically that the incidence of adjacent-segment compression fractures after PVP was much lower than in earlier reports [10]. Based on this information, we decided to test the hypothesis that PVP was contributing to the appearance of these compression fractures. We therefore injected the clinically relevant amount of bone cement into fractured cadaver vertebral bodies and studied the biomechanical changes in adjacent vertebrae under a physiological load (500 N).

## Materials and methods

### Selection of cadaver specimens

This study is a cohort interventional study. The presence of kyphosis, scoliosis, old fractures, and other abnormalities of the thoracolumbar cadaver specimens, determined by C-arm fluoroscopy, is excluded from the study. The remaining T12–L2 vertebrae then underwent measurement of their bone mineral density (BMD) with dual-energy X-ray absorptiometry (OSTEOCORE 3; Medilink, Mauguio, France). Specimens in which each vertebral body (T12–L2) had a BMD of < 0.75 g/cm^2^ were used for the experiment [11]. A total of 12 cadaver specimens met the above requirements. These embalmed cadavers [12] were provided by the Department of Human Anatomy, School of Basic Medical Sciences, Southern Medical University. The used cadavers were a donation for medical research.

### Establishing the fracture model

Because of the anatomy and biomechanical characteristics of vertebral bodies, compression fractures are most likely to occur on the upper one third of the vertebral body, close to the superior endplate. In patients with osteoporosis, these compression fractures are often accompanied by anterior cortical rupture of the vertebra. To achieve a model that was as close as possible to the clinical situation and ensure good repeatability of the experiment, we performed wedge osteotomy at the upper one third of the L1 vertebra and kept the superior endplate complete, thereby establishing a fracture model with previous literature completely followed [12, 13].

### Biomechanical laboratory equipment

An ElectroForce 3510 Material Testing Machine (Bose, Framingham, MA, USA) was used to complete the biomechanical test. Wintest control software, used to manage the test machine, can generate square waves and sine waves and can ramp, block, and perform high-precision data acquisition. We also used a GOM non-contact optical strain measurement system (Dom 3d Ltd., Shanghai, PRC) that consists of a Dell Precision T7600 computer workstation and Aramis measurement module application software (Aramis 6.3). This GOM non-contact optical three-dimensional deformation measurement system is mainly used for analyzing three-dimensional deformation and strain distribution of materials and components.

### Vertebroplasty instruments and bone cement

The Mendec® Spine percutaneous vertebroplasty system (Tecres, Verona, Italy) was used in this study. Bone cement was prepared in a ratio of 20 g powder/9.4 g liquid, wherein 20 g of the powder contains 67.5% (w/w) polymethylmethacrylate (PMMA), 30.0% (w/w) barium sulfate, and 2.5% (w/w) diphenylperoxide. The 9.4 g of liquid contains 99.1% (w/w) methyl methacrylate, 0.9% (w/w) *N*, *N*-dimethyl-*p*-toluidine, and 75 ppm hydroquinone.

### Vertebroplasty procedure

**Fig. 1:**
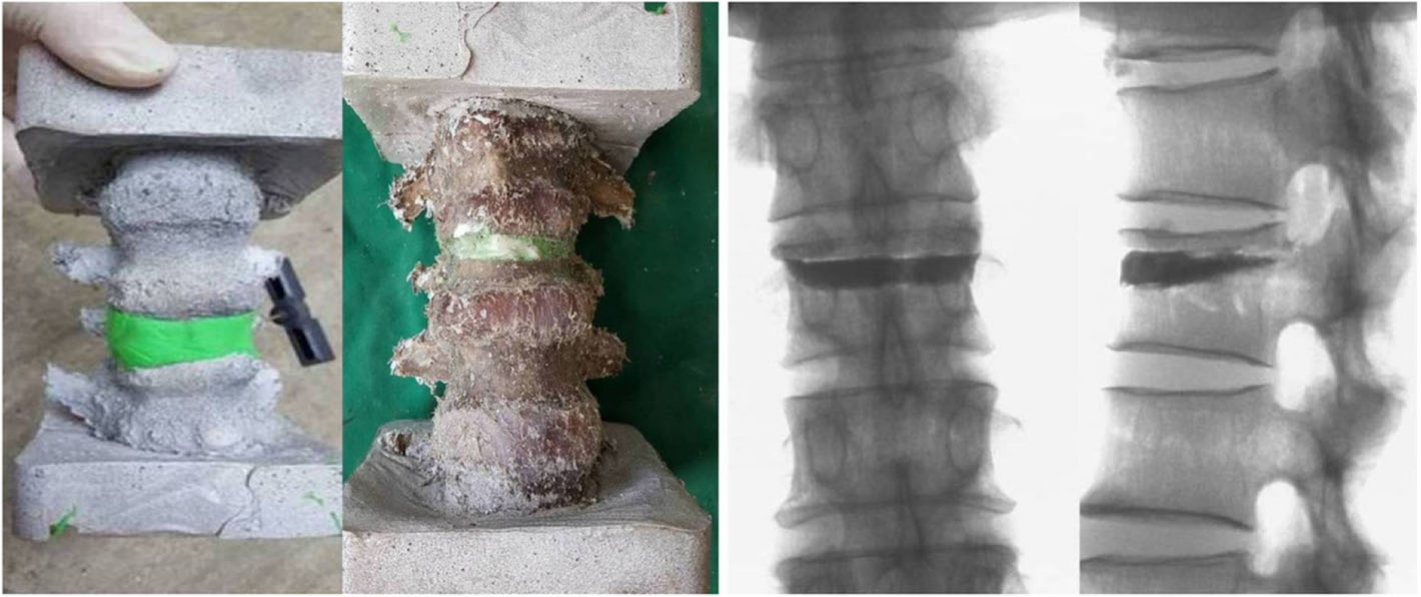
L1 fracture model was established, and bone cement was injected into the defect area of vertebral body

Biplane fluoroscopy was performed in all specimens. In each specimen, a 13-gauge needle was advanced to the osteotomy area of the L1 vertebral body via left pedicular puncture. The cement was prepared based on clinical operating standards, and the mixture was applied using an injector device. The volume of bone cement injected was based on filling fully the defective area without leakage (Fig. 1). Each amount of bone cement injected was recorded. Specimens were wrapped in briny gauze for 24 h, after which they were subjected to biomechanical testing.

### Specimen preparation and experimental measurements

After separating the T11–L3 vertebrae from the spinal segments, the intervertebral discs on the cranial and caudal sides were excised from T11 and L3, taking care not to damage the bony endplate. The paraspinal muscles were removed, retaining the anterior longitudinal ligament, intervertebral disc, articular process joints and joint capsules, spinous process, and the inter-spinous and spinous ligaments. The liquid was mixed with the PMMA powder at a ratio of 1.0:1.5, and the specimens were embedded at both ends (T11, L3) using an embedding mold (provided by Guangdong Provincial Key Laboratory of Medical Biomechanics, School of Basic Medical Sciences, Southern Medical University provided).

After confirming that the testing equipment was ready, the specimen was placed on the material testing machine (ElectroForce 3510). The weight was preloaded to avoid the effects of rheological properties, such as a time effect and relaxation and creep of the specimen. The maximum load was then set at 500 N with a loading rate of 5 N/s. The stiffness values during flexion, left lateral flexion, and extension were measured three times. The three measurements were then averaged, with that value used in the analyses. Using matte paint (black and white), the specimen was first treated on the front of the vertebral black background until the dark background was evenly covered. Then, the white paint was used for spotting to establish a GOM non-contact strain measurement system, which could be identified by the irregular speckled surface. We then waited for the surface paint to dry naturally.

**Fig. 2:**
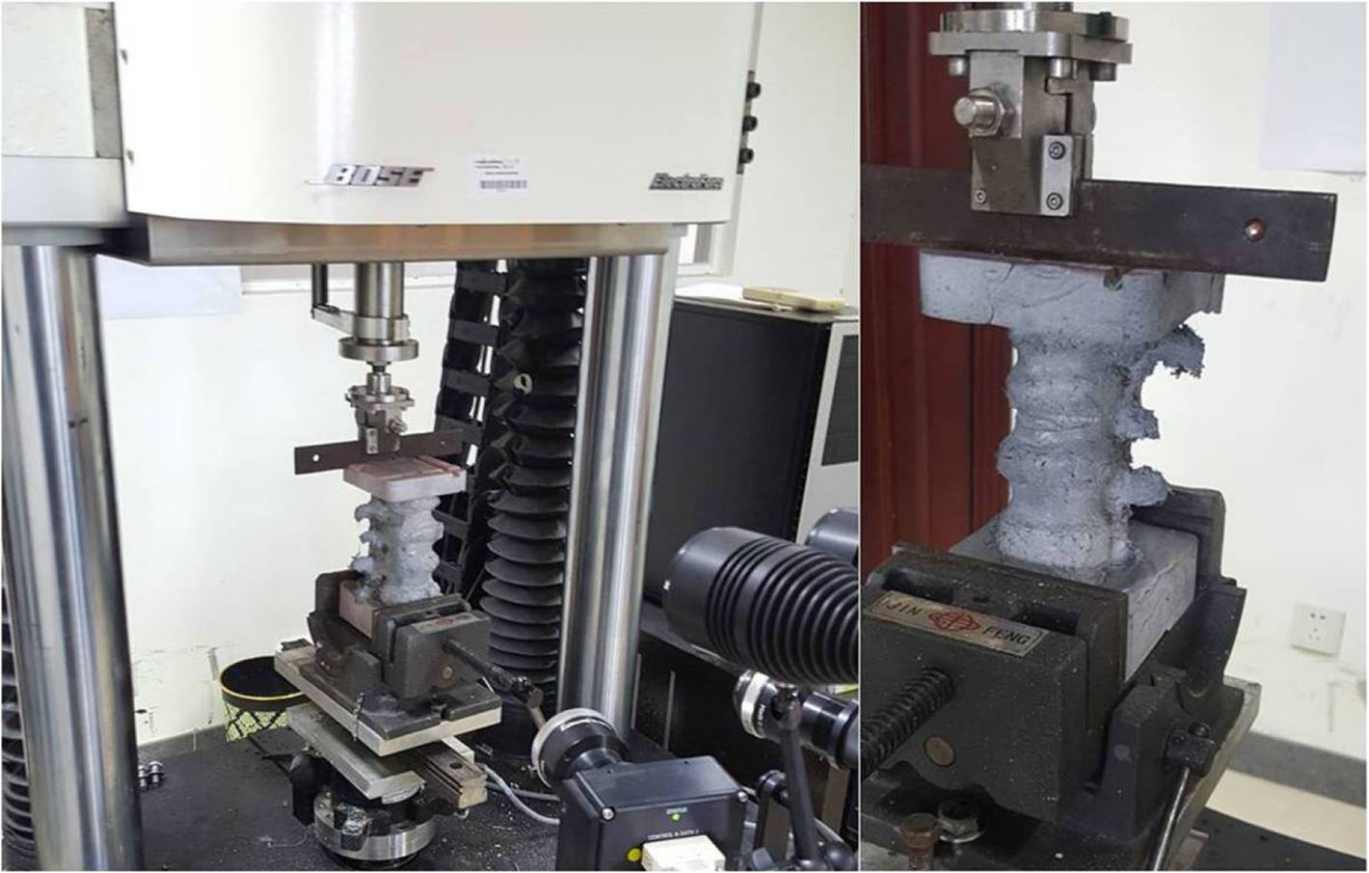
Specimens were placed on the mechanical test machine for testing

In general, the smaller the calibration plate, the greater is the accuracy of the measurement. In this study, based on the sizes of the specimens in this experiment, we selected a 55 × 44 mm^2^ calibration plate. The specimen was then placed on the loading table, and the bottom of the specimen was clamped with a vise. The GOM non-contact optical strain measurement system was then placed in front of the specimen. The fill light was opened, the specimen’s surface irregular spots were clearly recognized in the ARAMIS software control interface, and the movement range of the specimen was determined in the lens recognition range by appropriately moving the specimen. The strain of the specimen was then recorded in flexion, left lateral flexion, and extension up to a load of 500 N. In flexion and extension, the strain collection area was the front of the specimen, whereas in left lateral flexion the strain collection area was located on the left side of the specimen. After establishing the fracture model and performing vertebroplasty of the specimen, the above measurement process was repeated (Fig. 2).

### Statistical analysis

To avoid differences between specimens, a self-controlled comparison was performed for each specimen in the various states. Multiple-group comparisons were performed using one-way analysis of variance. If there was homogeneity of the variance, the LSD test was used to assess multiple comparisons. If there was unequal variance, the Dunnett-t3 test was used to assess multiple comparisons. All data were processed by SPSS 23.0 statistical software (SPSS, Inc., Chicago, IL, USA). A value of *p* < 0.05 was considered to indicate statistical significance.

## Results

The average amount of bone cement injected into the L1 vertebrae was 4.4 ml (3.8–5.0 ml). The BMDs of the specimens are shown in Table 1.

**Table 1:** Bone cement volume and bone mineral density of specimens

Specimen number	Injection volume of bone cement (ml)	BMD (g/cm^**2**^)		
		T12	L1	L2
1	4.5	0.292	0.278	0.234
2	4.8	0.417	0.403	0.409
3	3.8	0.525	0.656	0.540
4	4.0	0.423	0.442	0.423
5	4.8	0.471	0.470	0.458
6	4.5	0.447	0.474	0.486
7	4.0	0.437	0.501	0.515
8	3.8	0.484	0.669	0.706
9	4.5	0.678	0.706	0.772
10	4.0	0.742	0.737	0.694
11	5.0	0.715	0.728	0.718
12	4.8	0.687	0.654	0.712

### Intact stiffness of specimens

In flexion, intact stiffness was significantly lower after the fracture than before it (*p* < 0.05). Intact stiffness after augmentation was significantly restored (*p* < 0.05) but was still below the pre-fracture level (*p* < 0.05). In left lateral flexion, intact stiffness was significantly lower after the fracture than before it (*p* < 0.05). There was no significant difference in intact stiffness before and after augmentation (*p* > 0.05). In extension, although there was less stiffness after the fracture than before it, stiffness had been restored after augmentation, but the difference was not statistically significant (*p* > 0.05) (Table 2).

**Table 2:** Stiffness changes of specimens (N/mm, mean ± SD)

	Pre-fracture	Post-fracture	After augmentation	*F*	*p*
Flexion	242 ± 67	96 ± 24*	201 ± 65*^#^	22.001	< 0.001
Left lateral flexion	243 ± 117	122 ± 53*	182 ± 85	5.594	0.008
Extension	284 ± 155	241 ± 135	260 ± 147	0.262	0.771

*vs. pre-fracture, *p* < 0.05

^#^vs. post-fracture, *p* < 0.05

### Strain changes in the vertebral body

In flexion, T12 vertebral strain was significantly higher after the fracture than before it (*p* < 0.05). After augmentation, T12 vertebral strain was significantly lower than just after the fracture (*p* < 0.05), but augmentation did not result in any significant difference in strain from before the fracture (*p* > 0.05). In left lateral flexion and extension, there was no significant difference in strain among the three conditions (*p* > 0.05) (Table 3).

**Table 3:** Strain changes of vertebral bodies (%, mean ± SD)

		Pre-fracture	Post-fracture	After augmentation	*F*	*p*
T12	Flexion	0.15 ± 0.02	0.16 ± 0.05*	0.14 ± 0.01^#^	8.335	0.001
	Left lateral flexion	0.17 ± 0.05	0.19 ± 0.04	0.18 ± 0.06	2.660	0.085
	Extension	0.42 ± 0.09	0.37 ± 0.07	0.44 ± 0.08	0.041	0.673
L1	Flexion	0.08 ± 0.01	0.15 ± 0.01*	0.08 ± 0.01^#^	125.501	< 0.001
	Left lateral flexion	0.14 ± 0.01	0.16 ± 0.07	0.14 ± 0.02	0.943	0.400
	Extension	0.14 ± 0.01	0.12 ± 0.02*	0.46 ± 0.13*^#^	86.473	< 0.001
L2	Flexion	0.10 ± 0.03	0.20 ± 0.02*	0.13 ± 0.03*^#^	40.656	< 0.001
	Left lateral flexion	0.09 ± 0.01	0.22 ± 0.04*	0.11 ± 0.07^#^	28.279	< 0.001
	Extension	0.15 ± 0.02	0.09 ± 0.01*	0.13 ± 0.02*^#^	37.942	< 0.001

*vs. pre-fracture, *p* < 0.05

^#^vs. post-fracture, *p* < 0.05

In flexion, the L1 vertebral strain was significantly higher after the fracture than before it (*p* < 0.05). L1 vertebral strain was significantly less after augmentation than after the fracture (*p* < 0.05), but there was no significant difference from the pre-fracture strain (*p* > 0.05). In left lateral flexion, there was no significant difference between the three conditions (*p* > 0.05). In extension, L1 vertebral strain was significantly lower after the fracture than before it (*p* < 0.05). L1 vertebral strain after augmentation was significantly higher than either before or just after the fracture (*p* < 0.05) (Table 3). This change in extension might be related to vertebral cortical rupture after the fracture.

In flexion, L2 vertebral strain was significantly higher after the fracture than before it (*p* < 0.05). L2 vertebral strain was significantly less after augmentation than after the fracture (*p* < 0.05) but was significantly higher than it was before the fracture (*p* < 0.05). In left lateral flexion, L2 vertebral strain was significantly higher after the fracture than before it (*p* < 0.05). L2 vertebral strain was significantly less after augmentation than after the fracture (*p* < 0.05), but there was no significant difference from that before the fracture (*p* > 0.05). In extension, L2 vertebral strain was significantly less after fracture than before it (*p* < 0.05). L2 vertebral strain was significantly higher after augmentation than after the fracture (*p* < 0.05) but was lower than that before the fracture (*p* < 0.05) (Table 3).

### Strain changes in the intervertebral disc

In flexion, the T12/L1 intervertebral disc strain was significantly less after the fracture than before it (*p* < 0.05). T12/L1 intervertebral disc strain was significantly greater after augmentation than after the fracture (*p* < 0.05), but there was no significant difference from that before the fracture (*p* > 0.05). In left lateral flexion, T12/L1 intervertebral disc strain was significantly less after the fracture than before it (*p* < 0.05). T12/L1 intervertebral disc strain was significantly greater after augmentation than before or after the fracture (*p* < 0.05). In extension, there were no significant differences among the three conditions (*p* > 0.05) (Table 4).

**Table 4:** Strain changes of intervertebral discs (%, mean ± SD)

		Pre-fracture	Post-fracture	After augmentation	*F*	*p*
T12/L1	Flexion	1.18 ± 0.25	0.76 ± 0.29*	1.20 ± 0.16^#^	12.955	< 0.001
	Left lateral flexion	0.85 ± 0.67	0.72 ± 0.15*	1.79 ± 0.11*^#^	2.531	< 0.001
	Extension	1.23 ± 0.25	0.90 ± 0.43	1.29 ± 0.48	322.417	0.096
L1/2	Flexion	0.87 ± 0.07	1.25 ± 0.20*	0.89 ± 0.38^#^	8.959	0.001
	Left lateral flexion	1.00 ± 0.43	1.13 ± 0.24	1.51 ± 0.26*^#^	9.428	0.001
	Extension	1.02 ± 0.36	1.30 ± 0.49	1.07 ± 0.27	1.802	0.181

*vs. pre-fracture, *p* < 0.05

^#^vs. post-fracture, *p* < 0.05

In flexion, L1/2 intervertebral disc strain was significantly greater after the fracture than before it (*p* < 0.05). L1/2 intervertebral disc strain after augmentation was significantly less than that after the fracture (*p* < 0.05), but there was no significant difference from that before the fracture (*p* > 0.05). In left lateral flexion, L1/2 intervertebral disc strain was greater after the fracture than before it, but the difference was not statistically significant (*p* > 0.05). L1/2 intervertebral disc strain after augmentation was significantly greater than that either before or after the fracture (*p* < 0.05). In extension, there were no significant differences among the three conditions (*p* > 0.05) (Table 4).

## Discussion

The amount of bone cement injected into the vertebral body varied depending on the goal of the individual experiment. In most previous experimental studies, however, as much bone cement as possible was injected into the vertebral body, sometimes amounting to > 7 ml or even up to 10 ml. The bone cement completely filled the area between the upper and lower endplates of the vertebral body, in an “endplate-to-endplate” filling mode [12, 14, 15]. It was found that changes of the vertebral mechanical properties could lead to fracture of adjacent vertebrae [11]. Others, however, had different views. A small amount of bone cement could restore the stiffness of the vertebral body to its pre-fracture state, and too much bone cement injection could lead to vertebral stiffness beyond the pre-fracture level [16] and cause adjacent vertebral fractures [17]. Hence, filling the gap with too much bone cement was not a good choice. Clinical practice had confirmed that the volume of bone cement injected had nothing to do with the analgesic effect [18], so the spine surgeon in most cases did not need to fill the vertebral bone cement to endplate-to-endplate levels for fear of leakage. In general, injection of large dosage bone cement did not accord with clinical operation. These experimental results may not reflect the real situation. In the present trial, when the defect of the fractured vertebral body was filled with bone cement, the injection was stopped so that it was closer to the clinical situation.

Researchers found that [13], when bone cement was injected into vertebral bodies with compression fractures, the stress of adjacent vertebral bodies significantly increased, as did the intervertebral disc pressure. They thus speculated that this factor could be the biomechanical basis for adjacent vertebral fractures after PVP. Belkoff et al. found that restoration of the stiffness of the vertebral body in the thoracic and thoracolumbar regions required 4 ml [19]. With the deepening understanding of PVP surgery and the advances in biomechanical experiments, others believed that the new vertebral fractures after PVP were not caused by increased stiffness of the vertebral body. They indicated that it was the injected bone cement that caused vertebral body stiffness and slightly increased end-plate pressure—but not enough to lead to adjacent vertebral fractures [20, 21]. Although the strength of the vertebral body was greater after PVP than before fracture, and the stiffness was partially restored, it was still less than that before the fracture [22]. The results of this study are similar to the above literatures.

Biomechanical experiments and finite element studies had shown that the bone cement injection volume could affect recovery of the strength and stiffness of the fractured vertebral body, but there was no correlation with bone cement type [23]. To study the relations between the volume of the bone cement injection and recovery of the stiffness and strength of vertebral bodies in osteoporotic vertebrae, some researchers divided the bone cement volume into 2-, 4-, 6-, and 8-ml doses. In the thoracolumbar region, the strength was restored to 64% with 2 ml and to 100% with 8 ml. Stiffness was restored to 70% with 2 ml, to 94% with 6 ml, and to 116% with 8 ml [24]. Other researchers confirmed that 3.5 ml of bone cement could restore the normal stress distribution of the vertebral body. Returning to normal stiffness required 7 ml of bone cement [25]. In our previous clinical study [10], the average volume of bone cement was 3.6 ml, whereas in this experiment, the average volume of bone cement injected was 4.4 ml. This study found that the stiffness was partially restored after augmentation, but it could not be restored to the pre-fracture level. We also found that the strain of the adjacent vertebral body was increased after L1 fracture, although the increased in strain of the lower vertebral body (L2) was the most obvious change. After bone cement was injected into the fractured L1, the trend of strain change in the adjacent vertebrae was consistent. The strain of the upper adjacent vertebral body (T12) decreased to the pre-fracture level, whereas the strain of the lower adjacent vertebral body (L2) did not decrease to the pre-fracture level, remaining slightly higher than the pre-fracture level. This finding suggests that the injection of bone cement partially reversed the high strain state of the adjacent vertebral body after L1 fracture, which was consistent with recent biomechanical findings [26, 27]. Some researchers used double functional spinal units (FSUs) as their study object, similar to our study, but they did not establish a fracture model. They injected 10 ml of bone cement directly into the median vertebral body and found that load conduction of the anterior column had changed, which increased the risk of adjacent vertebral body fracture [15]. But we did not reach similar conclusions in our study. We speculated that, although the clinical dose of bone cement that was injected into the vertebral body of the fracture model could lead to local biomechanical changes, it was not sufficient to increase the risk of fractures in adjacent vertebral bodies [28]. This might explain why some clinical studies found that patients undergoing conservative treatment of an adjacent vertebral fracture were more likely to experience new fractures than those who had undergone PVP [6].

In addition, we could not ignore the intervertebral disc. In theory, the bone cement might increase the pressure on the adjacent disc, resulting in deformation of the adjacent endplate, causing the endplate and nearby cancellous bone to fracture. Thus, the results of stress and strain changes further exacerbated and ultimately led to adjacent vertebral fractures. Biomechanical studies found that deformation and fracture of the endplate may be the main mechanism of adjacent vertebral fractures [15]. Inferior endplate fractures were disproportionately common in adjacent vertebrae immediately above the treated level after PVP, potentially supporting a causative relation between vertebroplasty and adjacent vertebral fractures [29]. However, our results showed that injection of bone cement partially reversed the abnormal strain state of the adjacent disc after the fracture. The strain changes of the upper and lower intervertebral discs were inconsistent in this study, which may be related to cortical rupture of the fractured vertebrae. Similarly, a biomechanical experiment demonstrated that clinically relevant doses of bone cement injected into fractured vertebrae did not cause a concentration of endplate stress and hence did not damage adjacent vertebral bodies leading to vertebral fractures [30].

Some scholars suggested that augmentation of fractured vertebrae could be extended to adjacent levels (at risk of fractures) to maintain stiffness and strength, even preventing further fractures [31]. Thus, surgeons began to think about the feasibility of preventing bone cement injection into adjacent vertebrae. The finite element analysis study found that preventive PVP could reduce the risk of adjacent vertebral fractures, but it was possible to prevent fracture with at least a 20% filling rate in the adjacent vertebrae [9, 32]. As previous biomechanical studies had shown that prophylactic PVP could reduce or prevent the occurrence of adjacent segmental vertebral fractures [33], it was also thought that preventive PVP might be effective in clinical practice [7, 34]. Another biomechanical study, however, had shown that the risk of new vertebral fracture after PVP had not increased and suggested that preventive PVP might need to be treated with caution [35]. The present study also showed that clinical dosage of bone cement could reduce the high strain of adjacent vertebrae after fracture. So, PVP did not mean that the risk of adjacent vertebral fractures would increase. On the contrary, recent studies had found that early PVP might reduce the risk of adjacent vertebral fracture [36]. Clinical studies also confirmed that prophylactic PVP could not reduce the risk of adjacent vertebral body fracture, so it was not recommended [8]. Our results do not seem to support prophylactic vertebroplasty either.

In the past, it was thought, theoretically, that the mechanical changes in the upper and lower vertebral bodies and the intervertebral discs in biomechanical experiments were consistent and might be the basis for establishing a vertebral fracture model in some biomechanical experiments. The present trial, however, found that strain changes in adjacent upper and lower vertebral bodies and the intervertebral disc were not consistent. It may be related to cortical rupture of the fractured vertebrae? Or this is self-biomechanical properties which intervertebral disc plays a role? These questions also reflect the complexity of human biomechanical environment. Therefore, this result needs to be further researched and explored.

This study also has the following limitations. Because of the use of embalming cadaver specimens, formalin fixation has little effect on bone strength, but it can degrade the discs and ligaments, and the compression loading pattern may be different from that of fresh cadavers.

## Conclusion

Clinical dosage of bone cement could not completely restore stiffness to the pre-fracture level, but could partially improve the high strain state of adjacent vertebral bodies. The strain changes of the upper adjacent vertebrae and intervertebral discs were inconsistent with the lower adjacent vertebrae and intervertebral discs. PVP may therefore have partially reversed the abnormal strain state of adjacent vertebral bodies which was caused by fracture.

## Data Availability

There is no any other supporting data.

## Abbreviations

PVP: Percutaneous vertebroplasty
BMD: Bone mineral density
PMMA: Polymethylmethacrylate
